# Treatment of the railway freight wagon wash effluents by coagulation methods on accelator reactor

**DOI:** 10.1007/s40201-021-00695-w

**Published:** 2021-07-06

**Authors:** Sławomir Żak, Terese Rauckyte-Żak

**Affiliations:** Faculty of Chemical Technology and Engineering, University of Science and Technology, 3 Seminaryjna Street, 85-326 Bydgoszcz, Poland

**Keywords:** Railway freight wagon wash, Wastewater treatment, Accelator reactor, Two-stage coagulation, Pre-oxidation

## Abstract

This paper presents results of the research carried out on a system made to pretreat the effluents produced in water treatment of dirty surfaces of railway transportation means (RTMs) mainly G, H, T and incidental F classes of rolling stock according to the International Union of Railways (IURs). The installation was designed for coagulation–flocculation pretreatment of wastewater with flow accelator reactor (AR) in total amount of up to 75.0 m^3^ day^−1^. The raw wastewater (RW) was characterized by a significant diversity of loads: TSS (total suspended solids), TDS (total dissolved solids), COD & BOD_5_ (chemical & biochemical oxygen demand) and periodically it had extremely different colors, T_a_ (turbidity) and EE (etheric extract). The application of two-stage, coupled acid-alkali or alkaline-acid coagulation using aluminum coagulants with final flocculation and phase separation in the system implemented in practice to treat the wastewater of statistically typical composition, usually allowed to removal, accordingly: EE & TSS > 99% and to eliminate completely color and T_a_. However, COD and BOD_5_ were removal at different levels, depending on both initial concentrations and chemical composition of load pools registered in the RW, and a type of coagulation used. The use of pre-oxidation with aqueous solutions of hydrogen peroxide or peracetic acid coupled with coagulation based only on aluminum coagulants helps to achieve equal levels of removal of the basic indicator values and a sanitary clean stream of pretreated wastewater (PW) with a colony forming unit (CFU) of <100 ml^−1^.

## Introduction

RTMs for various cargo types such as: raw materials, semi-finished and finished products, participate in transferring huge masses and volumes of goods for use in three basic sectors of each state economy: manufacturing, processing and services, over the target distances [1*–*7]. This way of mass transportation is most often associated with a permanent change in transported cargos during the time and a significant change in a type and amount of residues on surfaces contaminated by transported goods [8*–*11]. Intensive use of transport fleets is inseparably connected with the necessity of cleaning dirty wagon surfaces and generation of wastewater loaded with the composition of pollutants as a function of the characteristics of transported materials [12*–*20]. An optimal organizational solution is a group cleaning of the transport fleet in a form of collective wagon washes to eliminate dispersed sources of wastewater and other wastes from the ones generated in the processes of wagon cleaning [21*–*28]. The character of washing facility operation must firstly take into account a class of rolling stock according to the division adopted by the IURs [29]. In case of wash facilities covering railway freight wagons of classes E, F, G, H, K, L, R and T, with the exception of rolling stock of classes I, Z and U, water washes not requiring special treatment conditions may be used. Then, the generated wastewater will mostly be characterized by significant differences in composition and concentration of TSS, T_a_, mainly caused by the presence of colloidal systems, the content of the sum of soluble mineral and/or organic substances TDS, as well as TC (total conductivity). COD & BOD_5_ and occasional color, as well as traces of oil, fat and/or oil derivatives included in the FOG (fats, oils & grease) index [8*–*11, 19*–*23] are also of less significance. Values of these parameters depend on the physical state, chemical composition, fragmentation, moisture content, heterophasic scale, packaging methods and tightness of packaging, as well as long-term repeatability of the category of transported materials [9, 11]. The composition and level of contamination load in the wastewater may also be a result of incidental mechanical damage to packaging that secures the materials during transport operations [11]. The total loads of contaminants disposed to the cleaning installations also include incidental microbiological and/or mycological infections, and parasitological infections, which in extreme cases, force the necessity of using periodical disinfection of the washed dirty surfaces of the rolling stock [30*–*33].

In literature, you can find mainly the records of methods of physicochemical pretreatment of the wastewater from such processes which consist in the application of coagulation–flocculation [34, 35] and electrocoagulation systems [36, 37]. Available process data and knowledge of physicochemical and/or biological basis of pretreatment and/or purification methods are quite limited.

The purpose of this work was to select a system to treat wastewater generated by water-based washing of railway rolling stock of G, H, T and incidentally F class on the designed and built installation, based on coagulation.

## Materials and methods

### Raw wastewaters

Generated RW was mostly a multiple, water-based dilution of component residues of transported freight masses (mainly G, H and T categories with incidental F category cases), characterized by significant changes in composition (Table 1) and load size. The pollution pool mainly consisted of unstable colloidal- and suspended polydisperse systems with a tendency to fast sedimentation, often with reduction properties (mostly a permanent decrease in rH (redox potential) value of wastewater kept during the time *–* 8*–*27 mV day^−1^ on average) with a tendency of rotting and coloring, which forced the necessity of their pretreatment.

**Table 1:** Characteristic of raw wastewaters (RW) and after detention and sedimentation (RW_(S)_)

No.	Parameter, unit	Range value for RW (median’s) ^a)^	Range value for RW_(s)_, (median’s) ^a, b)^
1	pH	6.4–8.8 (7.4)	6.2–8.2 (7.2)
2	Color (mgPt.Co l^−1^)	12–41 (27)	11–28 (18)
3	TDS (mg l^−1^)	286.4–1004.0 (462.4)	216.9–885.9 (437.8)
4	TSS (mg l^−1^)	263.2–1577.3 (948.9) 4011.6 ^c)^	89.6–211.2 (140.8)
5	COD (mg l^−1^)	293.3–1307.0 (424.5)	243.6–1004.9 (381.4)
6	BOD_5_ (mg l^−1^)	40.7–255.0 (148.1)	34.8–209.3 (103.3)
7	EE (mg l^−1^)	0.82–26.44 (10.26)	0.04–2.41 (0.72)
8	TN (mg l^−1^)	0.95–22.16 (4.60)	0.28–13.17 (3.39)
9	AN (mg l^−1^)	0.11–10.27 (3.66)	< 0.10–9.41 (2.95)
10	TP (mg l^−1^)	0.24–5.01 (2.63)	0.19–4.73 (1.56)
11	HMs, (mg l^−1^) ^d)^	0.23–4.66 (3.09) 7.64 ^e1)^	< 0.10–2.74 (1.92) 4.13 ^e2)^

where:

^a)^the median (m_1/2_) was determined basing on 187 measurement series over a six-month period for samples of RW taken at point A (Fig. 1) and for samples RW_(s)_ taken at point B (Fig. 1) after the retention in storage-averaging tank (1) and sedimentation;

^b)^the listed analysis results include cases where retention time of RW in section (1.1) of storage average tank (1) was determined as at least 90.0 ± 5.0 min and was not pumped into section (1.1) from intermediate tank (8) of the filtrate generated on filter unit (7) or a mixture of filtrate and water streams generated by backflushing the gravel filter water bed (4) (Fig. 1);

^c)^a single case of the wastewater generated from wagon washing after the transport of crushed rock aggregate;

^d)^the parameter heavy metals (HMs) includes the volumetric determination of the following elements: Cd, Cr_(T)_, Cu, Hg, Mn, Ni, Pb and Zn (no Cd, Cr_(T)_ were found) and Hg were found in any of the analyzed samples at levels exceeding the concentration threshold of 0.1 mg l^−1^);

^e1 and e2)^for this incidental value of determined HMs, the presence of the following individual metals (in mg l^−1^) was found in the stream of ^e1)^ RW: (Zn) 3.70, (Pb) 2.19, (Mn) 0.62 and (Cu) 1.33, and for the ^e2)^ RW_(s)_: (Zn) 2.37, (Pb) 1.06, (Mn) 0.56 and (Cu) 0.14, whereas the others (i.e., Cd, Cr_(T)_, Cu, Hg and Ni) did not exceed concentration levels of 0.1 mg l^−1^

### Experimental installation

The installation of a physicochemical treatment plant (PTP) with a daily capacity of up to 75.0 m^3^, in which the experiment was carried out, is presented in a simplified scheme in Fig. 1 [38]. RW from cleaning the transport surfaces of rolling stock, flowed gravitationally into a retention-average tank (1) and it was outflowed to its equalization and sedimentary section (1.1), where the composition was averaged and pollutants were separated in a water flux in a form of easily settling suspensions and randomly floating, insignificant volumes of light liquids (e.g. FOG). Then, they flowed through an overfall system to a pumping part (1.2) where a processing pump (1.3) pumped the processed wastewater to a pipe reactor (2) (RW_(s)_ marked flux). Under turbulent flow conditions, coagulant I^o^ from tank (9) and/or the coagulant II^o^ or neutralizing reagent (from tank 10) were dosed with dosing pumps (9.1) and (10.1) respectively, depending on the adopted option of one- or two-stage coagulation. The pipe reactor (2) equipped with process pH-meters at its inlet (pH 1) and outlet (pH 2), provided fast mixing and uniform distribution of chemical reagents before wastewater entering the processing volumes of a central, two-chamber reactor (3) of AR class. Before being injected into a fast mixing chamber (3.1) equipped with a slow-running frame mixer (3.2), 0.3% flocculent water solution was metered by a pump (11.1) from a preparation and dosing station (11). Then, the wastewater with formed flakes flowed through a deflector transfer system into the sedimentary chamber (3.3) of the AR, where, under the influence of gravitational forces, sedimentary separation and thickening of flocculated dispersed contaminant particles took place in the sedimentary pockets. During the next stage, the stream from the overflow system was directed to the process unit of an open, multilayer gravity filter (4), on which the residual colloidal and suspended fractions entrained by the water stream were stopped. PW as a filtrate was stored in a retention tank (5), from where it was fed by a pump set (5.2) to be reused in the cycle of cleaning the dirty surfaces of railway transport rolling stock (after being topped up with potable water (TW)) or discharged into the municipal sewer system. The developed installation also included the ability to disinfect wagons’ dirty surfaces by final washing with TW stored in tank (6) and pumped with pump set (6.1), after which, it was downstreamed with a water solution of peracetic acid or hydrogen peroxide, optionally metered into the pipeline in an appropriate proportion by a pump (12.2) from the station’ tank (12). There was also an option of disinfecting the gravel bed of filter (4) during back flushing with water from tank (5), additionally supplied with disinfectant pumped by pump (12.1) from the station tank (12). The wagon wash operation control software also included the option of pre-disinfection of the dirty surfaces of freight wagons before the physicochemical pretreatment stage, which was also tested in the PTP. The whole process system was equipped with a water supply unit (TW) in order to cover the losses caused by evaporation, the water remaining on the washed rolling stock surfaces; etc. (ca. 10% per batch of whole pre-cleaning cycle). In order to avoid excessive salinity of PW periodically, every 3*–*5 batches, the entire volume of used cleaning water was discharged into the sewer system. This volume was estimated on the basis of conductivity measurements *–* a limit value was established at the level of 2.5 mS/cm as salt discolorations often remained on dried surfaces for higher salt concentrations. In the treatment process of wastewater from railway rolling stock water cleaning, the waste flows were also generated in a form of:

- sediments accumulated in the sedimentation section (1.1) of the retention-average tank (1) *–* periodically removed (1*–*2 times a week) from the tank;
- sediments accumulated in the slow mixing chamber, in its sedimentary pockets of the sedimentary zone (3.3) of AR, which were periodically pumped by the sludge pump (3.4) into a dewatering unit, in a form of open gravity bag filters (7), where suspended solids were thickened using gravity forced filtration (the dewatered sediment was periodically removed), whereas a filtrate was directed to the start of the treatment system through an intermediate tank (7.1) and a pumping station (8);
- backflushing waters from the gravel filter backflushing process (4) *–* with a stream of pretreated water from tank (5) delivered by a pump (5.1) (the water backflushing of the filter was preceded by a purge of compressed air pumped by a blower (4.1). The used backflushing waters from the pumping station (8) were directed to the tank (1) at the beginning of the system with a pump (8.1). As an option with the gravel bed disinfection, the backflushing waters pumped from the tank (5) were additionally treated with water solutions of CH_3_COOOH or H_2_O_2_ metered with a pump (12.1) from station tank (12).

**Fig. 1:**
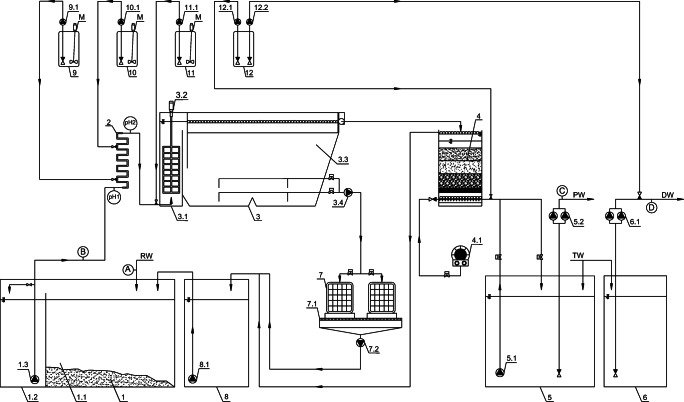
Simplified process flowchart of PTP; where: 1) retention-average tank for RW, 1.1) sedimentation section, 1.2) pump section, 1.3) process pump and bypass, 2) pipe reactor, 3) central process reactor of AR type, 3.1) quick mixing chamber, 3.2) slow frame mixer, 3.3) slow mixing chamber and sedimentary chamber with sedimentary pockets, 3.4) sedimentary pump, 4) open multi-layer gravel filter, 4.1) blower, 5) retention tank for PW, 5.1) bypass pump for averaging the composition of the pretreated stream and for backflushing the gravel filter (4), 5.2) pretreated water stream pump set for washing wagons or for discharging into the sewer system, 6) potable TW tank, 6.1) potable TW pump set, 7) gravity sludge dewatering station, 7.1) intermediate filtrate tank, 7.2) filtrate pump, 8) pumping station of wastewater from backflushing process of gravel filter (4) and of filtrate from sludge dewatering node (7), 8.1) pump of mixture of filter backflushing water and filtrate, 9) dosing station of coagulant (I^o^), 9.1) coagulant (I^o^) dosing pump, 10) dosing station for dosing coagulant (II^o^) or neutralization reagent, 10.1) dosing pump of coagulant (II^o^) or neutralization reagent, 11) flocculent solution preparation and dosing station, 11.1) flocculent dosing pump, 12) disinfectant dosing station, 12.1 and 12.2) disinfectant dosing pumps, DW *–* disinfectant water, A, B and C *–* sampling points for the analyses of RW, RW_(s)_ and PW, D *–* DW sampling point for control analyses, pH 1 and pH 2 *–* process pH-meters at the inlet and outlet of pipe reactor (2)

### Process reagents

Commercial coagulants of Kemira Kemipol were used for the research [39] for categories (I^o^) and/or (II^o^) of PIX®, PAX® and SAX® classes, the basic characteristics of which are given in Table 2.

**Table 2:** Basic characteristics of commercial aluminum and iron coagulants used for the research [39]

No.	Coagulant (basic composition)	Reaction, pH	Density, g l^−1^ at 20 °C	Metal content ^a)^, %
1	PIX® 113 (Fe_2_(SO_4_)_3_ in aqueous solution H_2_SO_4_)	< 1	1500*–*1570	11.0 ± 0.4
2	PIX® 116 (FeCl_3_ in aqueous solution HCl)	< 1	1310*–*1390	11.5 ± 0.5
3	PIX® 122 (Fe_2_(SO_4_)_3_ in aqueous solution H_2_SO_4_) ^b)^	< 1	1550*–*1570	12.6 ± 0.3
4	PAX® 16 (AlCl_3_ and polyaluminum chloride (Al(OH)_r_Cl_s_ + H_2_O (r + s = 3 where: 1.05 < r < 2) in aqueous solution HCl) ^c)^	< 1	1250*–*1280	8.2 ± 0.2 (Al_2_O_3_–15.5 ± 0.4)
5	PAX® 18 (AlCl_3_ and polyaluminum chloride (Al(OH)_r_Cl_s_ + H_2_O, (r + s = 3 where: 1.05 < r < 2) in aqueous solution HCl) ^d)^	1.0 ± 0.2	1350*–*1370	9.0 ± 0.3 (Al_2_O_3_–17.0 ± 0.6)
6	SAX® 18 (Na_2_Al_2_O_4_ in aqueous solution NaOH)	12.5 ± 0.5	1390*–*1510	9.5 ± 0.5 (Al_2_O_3_–18.0 ± 1.0) ^e)^

where:

^a)^coagulant metal content Al(III) or Fe(III); ^b) and c)^ free acid content (%): ^b)^ 3.0*–*4.0, ^c)^ 2.0*–*4.0; ^d)^ chlorides content (%): 21.0 ± 2.0, ^e**)**^ Na_2_O/Al_2_O_3_-based – 1.65-1.75 mol mol^−1^

The laboratory scale results were corrected and compared on a full scale installation using the algorithm of the control program for such doses of the tested coagulants that enabled us to obtain final pH < 9.0 and to use the same doses of flocculent (0.3% aqueous solution FLOPAM™ FO 4800 SNF Floerger) for all the tested coagulation variants (also using preliminary disinfection with aqueous solution 1.5% CH_3_COOOH (prepared from 15% (ρ = 1.1610 g ml^−1^) (ENVOLAB fine chemicals)) or 1.0% H_2_O_2_ (prepared from 30% (ρ = 1.1110 g ml^−1^) (ENVOLAB fine chemicals)). The established final pH value resulted from differences in the initial pHs of commercial coagulants and the concentrations of coagulant metal (Al(III) for PAX® group coagulants (16, 18) and Fe(III) for PIX® group coagulants (113, 116 and 122)) compensated by optimal volumetric doses of different initial pH, dosed into the pipe reactor (2). For the programmed final pH level of 8.0 ± 0.3, the maximum removal of indicator values was obtained at comparable concentrations of the introduced metal (M) of the tested commercial coagulant.

### Corrected by calculation volume

In order to compare the efficiency, the above mentioned concentration differences were eliminated by introducing the corrected by calculation volume (V_r_) per unit 1.0 m^3^ of treated wastewater according to the following relationships (a) and (b):

a $$ {\mathrm{V}}_{\mathrm{r}}={\mathrm{V}}_{\mathrm{exit}}\hbox{--} {\mathrm{V}}_{\mathrm{out}}, $$

b $$ {\mathrm{V}}_{\mathrm{out}}={\mathrm{V}}_{\mathrm{c}}+{\mathrm{V}}_{\mathrm{n}}+{\mathrm{V}}_{\mathrm{f}}\kern0.5em +{\mathrm{V}}_{\mathrm{ox}} $$

where:

V_exit_ *–* total volume at the system outlet (m^3^), V_out_ *–* sum of external volumes discharged into the treated wastewater (m^3^), including V_c_ *–* unit volume of coagulant solution (in two*–*stage coagulation options V_c_ = V_c(I)_ + V_c(II)_, where: V_c(I)_ *–* volume of coagulant (I^o^), and V_c(II)_ *–* volume of coagulant (II^o^)) (m^3^), V_n_ *–* unit volume of 7.5% aqueous NaOH solution for correction of reaction to final level within pH = 8.0 ± 0.3 (only in the option of single-stage coagulation with acidic coagulants) (m^3^), V_f_ *–* unit volume of 0.3% aqueous flocculent solution (m^3^) and V_ox_ *–* unit volume to be considered in the options with disinfection using aqueous solutions of CH_3_COOOH or H_2_O_2_ (m^3^).

The volume correction did not take into account the dilution effect of the filtrate generated on the filter unit (7) or the mixture of the filtrate streams and the streams generated by backflushing the gravel bed of the filter (4), as the tests were not performed under such conditions *–* no wastewater from the intermediate tank was pumped during the tests (8).

### Analytical part

In the collected, averaged samples of RW (point A in Fig. 1), after storage for sedimentation (RW_(s)_) in section (1.1) of the tank (1) (point B in Fig. 1) and at the outlet from the plant (PW) (point C in Fig. 1).

### Physicochemical parameters

In accordance with the applications given in the standards for water and wastewater, the following indicative values were determined (according to Polish Standards [40], APHA, AWWA and WEF [41]). The presence and concentrations of HMs such as Cd, Cr_(T)_, Cu, Ni, Mn, Pb and Zn were also checked and determined by FAAS method according to PN-ISO 8288:2002 and Hg method using cold vapour technique according to PN-EN 12338:2001 on AAS 700 Perkin Elmer apparatus (mineralization H_2_SO_4_ (ρ = 1.8420 g ml^−1^) *–* HClO_4_ (ρ = 1.6510 g ml^−1^) in 6:4 volume proportion). Hydrogen peroxide (iodometric method [42]) or peracetic acid (method described in positions [43, 44] and QUANTOFIX® Peracetic acid 500 and 2000 (Macherey-Nagel GmbH & Co. KG)) were determined in averaged samples of DW (point D on Fig. 1) from an autonomous process node. When the option of pre-disinfection with oxidants in the form of aqueous solutions of CH_3_COOOH or H_2_O_2_ was applied, the COD determination was corrected. Actual chemical oxygen demand was given after correction of this value by subtracting the share of residual hydrogen peroxide (from peracetic acid it is released according to simplified reaction scheme): 2CH_3_COOOH + 2H^+^ → 2CH_3_COOH + H_2_O_2_ [45] on the basis of COD_ω_ = COD_υ_ *–* φ‧Ψ (COD_ω_ *–* actual, COD_υ_ *–* determined in the post-reaction test, Ψ *–* H_2_O_2_ concentration in the test was determined by iodometric method [42], φ = 0.25 *–* correction coefficient adopted on the basis of data from the position [45–49]).

### Biological parameters

Periodical control tests were also applied for the general loading of RW and PW streams in accordance with the methodologies for the total number of microorganisms in 22 ± 2 °C after 72 h and in 36 ± 2 °C after 48 h according to PN-EN ISO 6222:2004, determining the level of CFU ml^−1^ parameter. A bacterial colony counter (LKNBTR-CHE-025 ADVERTI) was used for quantitative determinations. Moreover, in order to detect and identify bacteria that may appear in the tested samples of RW and/or PW, the following system analyses were used in the determinations: 1) *Clostridium* titre (incubation at 37 °C for 24*–*48 h in Thiogllycollate Broth at 37 °C). The presence of *Clostridium perfringens* was determined by screening on TSN Agar *–* incubation at 46 °C for 24 h under anaerobic conditions, 2) *Salmonella* and *Shigella* (preincubation at 37 °C for 18 h in Buffered Water), 2a) screening on Tetrathionate Broth *–* incubation at 43 °C for 24 h, 2b) screening on Selenite Broth *–* incubation at 37 °C for 18*–*20 h, 3) *Salmonella sp*. (screening for BPLS Agar) *–* incubation at 37 °C for 24 h, 3a) SS Agar *–* incubation at 37 °C for 24 h, 3b) Bismuth Sulf. Agar *–* incubation at 37 °C for 24 h, 3c) Pril Mannitol Agar *–* incubation at 37 °C for 24 h, medium: sodium and acid tetrationate with sodium selenite, discriminating-selective SS and Soltys *–* incubation at 37 and 43 °C, 3d) *Shigella sp*. (screening for SS Agar) *–* incubation at 37 °C for 24 h, XLC Agar *–* incubation at 37 °C for 24 h, Bismuth Sulf. Agar *–* incubation at 37 °C for 24 h, Pril Mannitol Agar *–* incubation at 37 °C for 24 h. *Salmonella* and *Shigella* were also identified by API 20 E (for *Enterobacteriaceae*). Detailed literature used for the development of the methodologies is given in [50–57]. Periodic parasitological evaluation was performed by means of microscopic analysis with the use of Delta Optical Genetic PRO Trino 40-1000x (4014066607) with 1600x option and Bresser MicroCam electronic eyepiece with a resolution of 5.0 million Pixels (maximum resolution 2592 × 1944 Pixels) and the application of basic calibration (measurement) slides with 1/10 mm micrometric graduation (Bresser). The presence of live intestinal parasite eggs *Ascarius sp*., *Trichuris sp*. and *Toxocara sp*. in the examined sample volumes was determined (after its initial concentration, flotation and centrifugation) by microscopic method using the indications given in [58, 59].

## Results and discussion

### One-stage coagulation

At the preliminary stage of this research [60], coagulation methods were selected, with the indication of double-stage coagulation to pretreat the tested wastewater. This approach was introduced by the results obtained in laboratory scale, proving unequivocally that the use of one-stage coagulation with acidic aluminum or iron coagulants with pH correction using 5.0 or 7.5% aqueous NaOH solution or 5.0% aqueous Ca(OH)_2_ solution does not allow us to obtain repeatability of levels of removal regarding basic indicator parameters within satisfactory limits for statistically typical loads of RW. The values of random distribution at the level of ±50% with reference to the value of median m_1/2_ determined for indicators in wastewater streams (PW_(s)_) were assumed in a standard way. The application of such systems in practice did not allow stable operation of the system measured by the efficiency and repeatability of the removal of contaminants. For example, the application of one-stage coagulation in the installation as the simplest variant with the use of iron coagulant PIX® 113 (acid solution Fe_2_(SO_4_)_3_) and neutralization with 7.5% aqueous NaOH solution led in statistically typical cases to the reduction of both TSS & EE >99%. However, it was possible only owing to the use of multilayer gravel filter (4) (Fig. 1). On the other hand, the removal of COD (48*–*67%), BOD_5_ (34*–*51%) (Table 3) was at different levels. For TDS, it was difficult to find an unambiguous and repeatable level of the removal of this parameter. Besides, the application of coagulation system (k) with neutralization (n) to correct the reaction (pH) resulted in an additional pool of measurable secondary external salinity (TDS_(k)(n)_) coming from the dosed purifying reagents. Moreover, for full scale tests on PW_(s)_ stream, the pretreatment option based on the constant dose of coagulant and neutralizing reagent, a color problem at the outflow often occurred, which was caused by the excess of Fe(III)-aquacomplexes in the pretreated water [61, 62]. The periodical excess of Fe(III) in relation to optimal doses and its coordination properties, which resulted in the generation of colored bonds from lemon yellow ((e.g. caused by the presence of chlorides ([Fe(Cl)_α_]^(α – 3)^ especially after the use of coagulant PIX® 116) and ([Fe(H_2_O)_β_]^3+^) through yellow caused by the presence of sulphates [Fe(SO_4_)γ]^(−2γ + 3)^ to red (e.g. caused by the presence of acetates [Fe(CH_3_COO)_2_]^+^ when peracetic acid was used, in the pre-disinfection option, where: α, β, γ *–* number of chloride, hydroxo or sulphate ligands respectively) [63–67]. In such a variant of coagulation treatment of stream RW_(s)_, it was necessary to overdose the acid coagulant (k) and to increase appropriately the dosage of neutralizing reagent (n) in order to ensure a certain repeatability of the removal levels of indicator values. It is inseparably connected with the increase in generated post-process sludge volume and the increase in secondary salinity (TDS_(k)(n)_) originating from dissociation and hydrolysis of soluble coagulant salt (k) and hydroxide *–* as a neutralizing reagent (n). In this process option, the external secondary salinity (TDS_(k)(n)_) may not be quantitatively compensated by sorption occurring on the precipitated floccules of dispersed contaminants and colloidal products of coagulant metal hydrolysis [68]. On the other hand, it may be compensated by the effect of dilution with supplementary water (ca. 10% of the process volume for a single batch of a complete treatment cycle). Such solution is a simple way to eliminate only dispersed pollutants (dispersion) in the aqueous phase, without any significant removal of pools of dissolved particle charges with an additional generation of secondary cationic-anionic salinity (TDS_(k)(n)_). Comparative studies to check if there are significant differences in the efficiency of PIX® 113, PIX® 116 and PIX® 122 coagulants showed that the differences in the removal levels of such parameters as COD, BOD_5_, TN, AN & TP (ammonium nitrogen & total phosphorus) were recorded at a small level of ca. 3*–*11%. It should be rather interpreted as a result of the nature of statistically acceptable differences resulting from, for example, random sampling or measurements themselves, especially in stage procedures of colorimetric methods while performing tests etc. Significant differences were found in color – when PIX® 116 was used, periodically higher values of this parameter were recorded at the outflow by approx. 20*–*30% (the effect of formation of ([Fe(Cl)_α_]^(α – 3)^ type complexes with the excess of free forms of Fe^3+^‧aq) than when PIX® 113 and PIX® 122 were used, because they contain sulphate salts [62, 69] which resulted from a different mobility and ionic strength of chlorides (PIX® 116) and sulphates (PIX® 113 and 122) [62, 70]. Comparable levels, respectively 75 and 60% determined in stream PW_(s)_ were obtained for COD and BOD_5_, but not exceeding removal.

**Table 3:** Removal levels (%) or change of selected parameters of PW using coagulation with PIX® 113 and neutralized with the solution of 7.5% NaOH ^a – c)^

No.	Parameter	% removal or change		
		Minimum value	Maximum value	Value of the median’s ^d)^
1 1.1 ^g)^	pH	7.9 7.7	8.8 8.7	8.3 8.2
2 2.1 ^g)^ 2.2 ^g)^	Color	73 82 57	89 95 88	84 91 79
3 3.1 ^g)^ 3.2 ^g)^	TDS ^e)^	7 5 5	15 13 13	10 8 7
4	TSS ^f)^	tr ^f1)^	tr ^f1)^	tr ^f1)^
5 5.1 ^g)^ 5.2 ^g)^	COD COD_ω_^h1)^ COD_ω_^h2)^	48 46 39	67 64 57	55 52 48
6 6.1 ^g)^ 6.2 ^g)^	BOD_5_	34 36 29	51 50 44	41 42 35
7	EE ^i)^	tr	tr	tr
8 8.1 ^g)^ 8.2 ^g)^	TN	12 9 11	24 19 22	17 14 17
9 9.1 ^g)^ 9.2 ^g)^	AN	5 3 4	12 9 11	8 7 7
10 10.1 ^g)^ 10.2 ^g)^	TP	74 75 71	91 88 85	84 81 78
11	HMs ^i)^	48	86	73

where:

^a)^results are presented here for operational conditions where removal was determined based on measurements at points B and C (Fig. 1) excluding incidental exceedances of the limit neutralization reaction level (pH) given below in reference ^b)^ and described in the text below;

^b)^the process was carried out with the control of uniformity of mixing the reagents using the process pH-meters at the inlet (pH 1) and outlet (pH 2) of the pipe reactor (2) by determining the dose (k) of coagulant PIX® 113 in the dosing mode “to pH” with respect to the algorithm of pH-meter indication (pH 1) and the dose (n) of aqueous solution of 7.5% NaOH with reference to the dose of PIX® 113 coagulant in programmed relationships, with reaching calculated value of pH_(PIX 113)_ = 1.2‧pH_(7.5% NaOH)_ respectively, but with keeping the additionally set upper threshold value after the neutralization at pH = 8.8;

^c)^the retention time of the effluents (the total flow time) in the process chambers (3.1) and (3.3) of the AR (Fig. 1) for the procedures of experimental series was set at the level 90.0 ± 3.0 min;

^d)^the median (m_1/2_) in % of the removal levels (or the parameter change rate) determined basing on measurement series, for which the ranges of parameter values of the incoming wastewater are given in Table 1;

^e)^it is given here the level of removal determined by the relationship: η_(TDS)_ = {[1 – ((TDS_(RW(s))_ – TDS_(PW)_ + TDS_(k)(n)_)/TDS_(RW(s))_)]}‧100% (where: η_(TDS)_ – the level of removal of TDS parameters in % determined at the outlet – the samples taken at point C (Fig. 1), TDS_(RW(s))_ – the inlet level the samples taken at point B (Fig. 1) and TDS_(PW)_ – TDS the load pool removed by coagulation (k) and neutralization (n), TDS_(k)(n)_) – the pool of external load carried in with (k) PIX® 113 coagulant and (n) neutralizing reagent (7.5% NaOH);

^f)^the use of multilayer gravel filter (4) (Fig. 1) results in PW and full removal of dispersed phases (TSS & EE) and T_a_ parameter, also in case of preliminary disinfection testing with 1.0% H_2_O_2_ or 1.5% CH_3_COOOH (where ^f1)^: tr – total removal);

^g)^No. 1.1, 2.1, 3.1, 5.1, 6.1 and 8.1 to 10.1 series of determinations (12 series) after initial disinfection of rolling stock surfaces using 1.0% solution H_2_O_2_ and then typical aqueous treatment washing and No. 2.2, 3.2, 5.2, 6.2 and 8.2–10.2 series of determinations (15 series) after initial disinfection of rolling stock surfaces using 1.5% CH_3_COOOH and then typical washing (HMs parameter was not analyzed for these cases);

^h1 and h2)^for samples with pre-disinfection ^h1)^ 1.0% H_2_O_2_ or ^h2)^ 1.5% CH_3_COOOH COD parameter was given on the basis of the relation: COD_ω_ = CODυ – φ‧Ψ;

^i)^the use of a multilayer gravel filter (4) (Fig. 1) resulted in a clear effluent and a complete removal of dispersed phases (TSS & EE) and the T_a_ parameter, also when pre-disinfection is tested with 1.0% H_2_O_2_ or 1.5% CH_3_COOOH (where: tr – total removal)

### Oxidant effect

The application of pre-oxidation before the coagulation treatment stage in this technological variant did not lead to any significant improvements in the parameters of PW at the outlet of the installation, but it often complicated considerably the process, especially after the application of aqueous H_2_O_2_ solution (Tables 3 and 4. No. 2.1, 3.1, 5.1, 6.1, 8.1–10.1). This was probably due to the occurring residual processes based on catalytic reactions involving mainly Fe(III)-aquacomplexes, Fe(III)-hydroxycomplexes (e.g. [FeOH]^2+^, [Fe(OH)_2_]^+^ and [Fe_2_(OH)_2_]^4+^ [71, 72]) as well as Fe(III)-hydroxy-peroxycomplexes (Fe^III^(HO_2_)^2+^ and [Fe^III^(OH)(HO_2_)]^+^ [73, 74]). This resulted in H_2_O_2_ decomposition according to a number of radical, radical-ionic and ionic reactions, known from the literature, e.g. [71, 72, 75–77]: Fe^3+^ + H_2_O_2_ ↔ Fe^III^(HO_2_)^2+^ + H^+^; Fe^III^(HO_2_)^2+^ + H_2_O_2_ → Fe^2+^‧aq + HO_2_^•^, Fe^III^(HO_2_)^2+^ + H_2_O_2_ ↔ Fe^III^(OH)(HO_2_)^+^, Fe^III^(OH)(HO_2_)^+^ → Fe^2+^‧aq + HO_2_^•^ + OH^−^, Fe^3+^‧aq + HO_2_^•^ → Fe^2+^‧aq + O_2_ + H^+^, Fe^3+^‧aq + HO^•^ → FeOH^3+^ → FeO^2+^‧aq + H^+^, 2Fe^3+^‧aq + H_2_O_2_ → 2Fe^2+^‧aq + O_2_ + 2H^+^ etc. During these transformations, the reactions (at least at the residual level), which are described in the literature as Haber-Weiss or Haber-Willstätter systems, and a whole series of reactions initiated by the presence of Fe(II) iron, typical for the transformations of Fenton system, e.g. [76, 78–84], have certainly had a significant role: Fe^2+^‧aq + H_2_O_2_ → Fe^3+^‧aq + HO^•^ + OH^−^, Fe^2+^‧aq + H_2_O_2_ → FeO^2+^‧aq + H_2_O, FeO^2+^‧aq + H_2_O_2_ → Fe^2+^‧aq + O_2_, and the reaction of Fe(II) with radical products, Fe^2+^‧aq + HO^•^ → Fe^3+^‧aq + OH^−^, Fe^2+^2027aq + HO_2_^•^ → Fe^3+^‧aq + HO_2_^−^ etc. The effect of very probable parallel course of these side reactions to the coagulation (k) was significant foaming, taking place on the surface of the process volume (3.1) of AR chamber (3). It also induced an increase in color and turbidity levels, as well as a simultaneous worsening of separation process of sedimentation of precipitated and flocculated phases in the volume of process chamber (3.3). It was also manifested in a complete disappearance of an irregular phase of the suspended bed (upflow sludge blanket), at 3.3*–*4.7 m h^−1^ of flow rate through this volume, resulting in a decrease in quality of the wastewater directed onto the filtration stage, which forced the necessity to backflush the filter (4) more frequently and it also included an increase in the total volume of backflush wastewater directed into the intermediate tank (8).

**Table 4:** Removal levels (%) of selected parameters of the PW by means of double-stage coupled acid-alkali coagulation using (I^o^) PIX® 116 – (II^o^) SAX® 18 system ^a)^

No.	Parameter	% removal or change		
		Minimum value	Maximum value	Value of the median’s ^b)^
1 1.1 ^f)^	pH	8.1 8.2	8.8 8.7	8.3 8.1
2 2.1 ^f)^	Color	79 66	94 91	91 86
3 3. 1 ^f)^	TDS ^c)^	6 6	15 11	11 9
4	TSS ^d)^	tr ^d1)^	tr ^d1)^	tr ^d1)^
5 5. 1 ^f)^	COD COD_ω_^g)^	49 43	69 72	59 59
6 6. 1 ^f)^	BOD_5_	38 32	52 47	43 39
7	EE ^d)^	tr	tr	tr
8 8. 1 ^f)^	TN	11 9	31 25	24 21
9 9. 1 ^f)^	AN	7 6	18 14	11 11
10 10. 1 ^f)^	TP	78 75	89 84	84 78
11	HMs ^e)^	68	87	81

where:

^a)^(I^o^): acid coagulant PIX® 116, (II^o^): SAX® 18 alkaline coagulant according to the data of the safety data in Table 2. Doses of coagulants were introduced into the pipe reactor (2) (Fig. 1) in the “up to pH” mode to control the uniformity of mixing the reagents using process pH-meters at the inlet (pH 1) and outlet (pH 2). In this dosing mode, the first-dose coagulant was dosed in relation to the pH-meter indication algorithm (pH 1) at the reactor inlet (2) and the second-dose coagulant was regulated in relation to the first-dose coagulant at pH_(PIX 116)_ = 1.2‧pH_(SAX 18)_, respectively, but the programmed limit of pH after the second coagulation stage was maintained at the level not exceeding pH = 8.8;

^b)^m_1/2_ – median (½ order) determined on the basis of 22 series of measurements carried out over two quarters, maintaining the retention time in process volumes (3.1) and (3.2) (Fig. 1) analogous to those given in reference ^c)^ Table 3, determining the removal on the basis of measurements in points B and C (Fig. 1) and at the level of determined values of RW parameters within the limits given in Table 1;

^c)^the removal level for stream RW_(s)_ is given in reference ^e)^ to Table 3;

^d)^the use of a multilayer gravel filter (4) (Fig. 1) resulted in a clear effluent (full removal of T_a_) and of total dispersion phase removal (EE & TSS), even after tests with 1.0% H_2_O_2_ pre-disinfection (where ^d1)^: tr – total removal);

^e)^the sum of HMs: Cd, Cr_(T)_, Cu, Hg, Mn, Ni, Pb, Zn – this parameter was taken into account when the concentration of any of them exceeded the level of 0.1 mg l^−1^ – such concentrations were found in 14 samples taken (determined as m_1/2(HMs)_ = 1.69 mg l^−1^) from 22 process batches (no concentrations of Cd, Cr_(T)_ and Hg exceeding the level of 0.1 mg l^−1^ were found in any of the samples);

^f)^the series of determinations (14 series) after the initial disinfection of rolling stock surfaces using 1.0% H_2_O_2_ and then typical washing (HMs parameter not analyzed in these cases);

^g)^COD parameter is presented as COD_ω_ based on the relation given in footnotes ^h1)^ to Table 3

### Double-stage coagulation

The application of double-stage coagulation based on iron (III) and aluminum (III) coagulants, such as PIX® 116 – SAX® 18 system, did not lead to any significant increases in removal levels of analyzed wastewater parameters (Table 4) in relation to PIX® 113–7.5% NaOH system (Table 3). The obtained removal levels were comparable, but the volume and dry mass of post-coagulation sludge increased significantly (ca. 15*–*35%), which was mainly a function of the increase in the share of polynuclear hydrolysis products initiated by the intermediate ionic forms such as Al_13_O_4_(OH)_24_^7+^ [69, 85] and/or Al_8_(OH)_20_^4+^ [86].

Pairs of reagents at flow rates 2.8–4.1 m h^−1^ for PAX® 16 – SAX® 18 and 2.2–3.5 m h^−1^ for PAX® 18 – SAX® 18, in chamber (3.3) allowed producing an irregular and suspended layer in the presence of the residual amounts of particles that did not flocculate and settle due to their size and the level of linear flow rate. The presence of this specific suspended bed (sludge blanket) additionally improved the clarity of PW directed to another process node (4) (Fig. 1).

The application of double-stage coagulation with aluminum reagents additionally induced co-precipitation, which also included dissolved natural organic matter, manifested by the increase in the removal of dissolved loads, not to be obtained using the single-stage coagulation variant with neutralization [62]. This co-precipitation as the method to increase the elimination level of soluble contamination load is inseparably connected with the polynuclear hydrolysis of aluminum products with a dominant share of crystalline or amorphous structures and their mutual mixed forms, generated after exceeding the thresholds of their precipitation during neutralization and a parallel second degree of coagulation connected with neutralization. Then, during the formation of floccular structures, a specific closure of soluble impurities and nanodispersion occurs, which results in a measurable increase in removal of load, especially COD and BOD_5_ [62, 69, 73, 74, 85–91]. In the variant of double-stage coagulation based on aluminum (III) salts, of the initiation of these phenomena is connected with the formation of increased volume of sediments with a significantly developed sorption surfaces also enabling measurable removal of salinity caused by labile anionic or cationic forms or their coordination combinations being a part of the total salinity load pool [62, 68, 71]. A side effect of these processes may be partial sorption of the pool of dissolved substances on surfaces of products hydrolysis of coagulants and co-precipitation processes.

### Oxidative effect

Application of preliminary pre-oxidation of the wastewater, especially using H_2_O_2_, does not lead to an increase in the amount of troublesome sludge treated wastewater and does not cause higher secondary salinity. However, its use in the case studied here should be considered only for the use of coupled coagulation based on aluminum coagulants, e.g. PAX® 16 (PAX® 18) – SAX® 18, etc. Then, for such combinations of coagulants, you will avoid: 1) destructive catalytic activity of Fe(III), occuring after the application of the systems with PIX® reagents with reference to oxidizing disinfectants (CH_3_COOOH or H_2_O_2_) and 2) adverse secondary quality effects (e.g. salinity (TDS_(ox)_)) being a result of mineralization of the organic load by the introduced oxidants. In full technological scale, using the double-stage coagulation coupled with aluminum salts (PAX® 16 (or PAX® 18) – SAX® 18 or SAX® 18 – PAX® 16 (or PAX® 18)), no significant effect indicator reagents used for pre-oxidation on the removal level of contaminants in the tested wastewater was found and the results were at the level obtained during pretreatment without pre-oxidation (Table 5). Basic components of aluminum coagulants: aluminum and polyaluminum chloride in acid coagulants (PAX® 16 and 18) and sodium aluminate in alkaline coagulant (SAX® 18), in the environment of tested wastewater were chemically inert towards H_2_O_2_, without causing its decomposition or blocking, e.g. by coordination.

**Table 5:** Comparison (%) of removal levels of selected parameters for PW with double-stage coagulation in acidic and alkaline (I^o^*–*II^o^) ^a, c)^ and alkaline-acidic options (II^o^*–*I^o^) ^b, c)^ using aluminum coagulants

No.	Parameter	% removal or change ^d)^		
		Minimum value (I^o^*–*II^o^) (II^o^*–*I^o^)	Maximum value (I^o^*–*II^o^) (II^o^*–*I^o^)	Value of the median’s ^e)^ (I^o^*–*II^o^) (II^o^*–*I^o^)
1	pH ^c)^	7.9 7.8	8.7 8.6	8.2 8.1
2	Color	83 92	tr tr	tr tr
3	TDS ^f)^	5 7	11 15	8 10
4	TSS ^g)^	tr ^g)^ tr ^g)^	tr ^g)^ tr ^g)^	tr ^g)^ tr ^g)^
5	COD	54 49	61 69	61 64
6	BOD_5_	37 41	54 51	42 43
7	EE ^h)^	tr tr	tr tr	tr tr
8	TN	13 15	27 31	19 24
9	AN	9 12	19 24	14 12
10	TP	81 89	93 95	90 91
11	HMs ^h)^	75 66	84 89	77^h1)^ 81^h2)^

where:

^a and b)^I^o^ (or II^o^): acid coagulant PAX® 18 and II^o^ (or I^o^): basic coagulant SAX® 18 according to the data in Table 2. The notations (I^o^*–*II^o^) and (II^o^*–*I^o^) denote the sequence options for dosing into the continuous flow pipe reactor (2) (in Fig. 1) of coagulant doses introduced in “up-to-pH” mode to control the uniformity of mixing the reagents using process pH-meters at the inlet (pH 1) and outlet (pH 2). In such dosing mode, the first-stage coagulant dose (I^o^) was metered according to pH-meter indication algorithm (pH 1) at the reactor inlet (2), whereas the second-stage coagulant dose (II^o^) was regulated by program with reference to the first-stage coagulant dose according to the set relation: pH_(PAX 18)_ = 1.2‧pH_(SAX 18)_ or pH_(SAX 18)_ = 1.2‧pH_(PAX 18)_, respectively but not exceeding the upper limit of pH after the second stage pH = 8.8;

^c)^the effluents retention time (the total flow time) through the process chambers (3.1) and (3.2) of the AR (Fig. 1) was determined for the procedures of this experimental series at the same level as given in footnote ^c)^ to Table 3;

^d)^the parameters of RW directed onto the test installation do not exceed the limit values and are within the ranges of values given in Table 1;

^e)^the median (order ½ (m_1/2_)) determined on the basis of 19 measurement series for the coagulation option (I^o^-II^o^) and 20 ones for the coagulation option (II^o^-I^o^);

^f)^is the removal level determined using the same relationship as in ^e)^ of Table 3;

^g)^removal levels guaranteed by additional protection in a form of final filtration stage as the unit of multi-layer gravel bed (4) (Fig. 1) similarly to the other tested coagulation options (where ^g)^: tr – total removal);

^h)^the value of the determined sum of HMs (where HMs: Cd, Cr_(T)_, Cu, Ni, Pb and Zn) is given for the individual process batches, when the concentrations of individual recorded in the RW exceeded level 0.1 mg l^−1^ and such levels were found for ^h1)^ in 10 (determined as m_1/2(HMs)_ = 1.66 mg l^−1^) and for ^h2)^ in 9 (determined as m_1/2(HMs)_ = 2.89 mg l^−1^) process batches (no Cd, Cr_(T)_, Hg, and Ni concentrations exceeding 0.1 mg l^−1^ were found in any sample)

In practice, the pre-disinfection using H_2_O_2_ may be considered, but using such doses that result in limited pre-coagulation. At the same time, this stage and the decomposition resulting from the complex disinfection activity exhausts the mass of the oxidant circulating without its level of free unused concentration directed to the subsequent treatment stages. Such a solution, with permanently changed parameters of treated RW, will make the use of system significantly more difficult, which will be related to the necessity of current analytical assessment of H_2_O_2_ demand for the purposes of disinfection and pre-coagulation.

### Biological hazard

Based on literature data [92*–*95], it was assumed that potential germs, which may periodically occur in the generated wastewater, in a predominant number of units, will not be isolated in single units but will be mainly grouped in particles, clumps or lumps of biologically active colloidal-suspended fractions and surrounded by substances protecting them. It was found that substances accompanying pathogenic microorganisms formed with them, e.g. in aggregates, suspended solids or larger particles of pollutants, which will not be coagulated and/or flocculant condensed and settled to the sludge accumulated in pockets (3.4) of AR, can be effectively filtered out. In the case of free-floating microorganisms with an external structure that prevents their coagulation and/or flocculation, the use of CH_3_COOOH or H_2_O_2_ as pre-oxidation is provided for. Applying these reagents as chemicals was aimed at causing destruction and modification of external structures of free-floating pathogenic organisms by oxidation and pre-coagulation accompanying the pre-disinfection and then a complementary coagulation and/or flocculation in the process volumes of AR, leading to eliminate these organisms maximally. The residual amounts of them were eliminated in the volume of the filtration bed of the process unit (4). This way, filtration was an important initial step for the disinfection of the pretreated stream of effluents intended for reuse. In addition, to eliminate risks associated with the development of a biological membrane containing pathogens originated from the RW, an optional disinfection of the filter gravel bed (4) was provided for backflushing with water containing CH_3_COOOH or H_2_O_2_ water, supplied from tank (12) using a separate membrane pump (12.2) with an individual adjustment of metering the disinfectant. This procedure was applied incidentally and preventively, due to a lack of risk of the occurrence pathogenic microbial infections of the PW. The level of CFU determined at 22 °C after filtration (in samples collected at point C in Fig. 1) was recorded for each sample significantly below the threshold value of 100 CFU ml^−1^. No pathogenic bacteria of the genera *Clostridium perfringens*, *Salmonella sp*. and *Shigella sp*. was found in the samples of RW and PW collected for periodic evaluation. Microscopic analysis also did not reveal the presence of helminth spores of genera grouped in ATT parameter (*Ascaris sp*., *Trichuris sp*. and *Toxacara sp*.) and cysts and/or trophozoites of *Cryptosporidium parvum*, *Giardia lamblia* or *Entamoeba histolytica* were not identified. During the research, the problem of pathogenic mycological and parasitological loads did not occur. However, it does not mean at all that it will not be significant even at the levels of irregular incidental short or long-term infections, which requires protection in form of filtration and disinfection procedures.

## Conclusions

1. The continuous flow installation based on an accelator type two-chamber reactor is an appropriate technical solution enabling effective pretreatment and the reuse of sanitary safe, treated wastewater resulting from washing dirty surfaces of railway rolling stock of class G, H, T and, incidentally, class F.
2. The highest efficiency measured by the removal level of indicator values such as TSS, EE, color, COD and BOD_5_ and satisfactory repeatability of removal is obtained by using double-stage, coupled acid/alkali or alkaline/acid coagulation with the use of aluminum coagulants in coagulation pairs, e.g. PAX® type (16 or 18) and alkaline SAX® type 18 coagulants with a final flocculation and gravity phase separation and a complementary filtration under continuous flow conditions.
3. When pre-oxidation with aqueous solutions of peracetic acid or hydrogen peroxide is used, coupled coagulation based only on aluminum coagulants, e.g. PAX® 16 – SAX® 18 or SAX® 18 – PAX® 18, with the help of which it is possible to achieve equal levels of removal of the basic indicator values and a sanitary clean stream of pretreated water with a CFU of <100 ml^−1^.
